# Lysing bloom-causing alga *Phaeocystis globosa* with microbial algicide: An efficient process that decreases the toxicity of algal exudates

**DOI:** 10.1038/srep20081

**Published:** 2016-02-05

**Authors:** Guanjing Cai, Xujun Yang, Qiliang Lai, Xiaoqi Yu, Huajun Zhang, Yi Li, Zhangran Chen, Xueqian Lei, Wei Zheng, Hong Xu, Tianling Zheng

**Affiliations:** 1 State Key Laboratory of Marine Environmental Science and Key Laboratory of the Ministry of Education for Coastal and Wetland Ecosystems, School of Life Sciences, Xiamen University, Xiamen 361005, China; 2 Key Laboratory of Marine Biogenetic Resources, Third Institute of Oceanography, State Oceanic Administration, People’s Republic of China

## Abstract

Algicidal microbes could effectively remove the harmful algae from the waters. In this study, we were concerned with the ecological influence of an algicide extracted from *Streptomyces alboflavus* RPS, which could completely lyse the *Phaeocystis globosa* cells within two days. In microcosms, 4 μg/mL of the microbial algicide could efficiently remove *P. globosa* cells without suppressing other aquatic organisms. Bioluminescent assays confirmed that the toxicity of microbial algicide at this concentration was negligible. Interestingly, the toxicity of *P. globosa* exudates was also significantly reduced after being treated with the algicide. Further experiments revealed that the microbial algicide could instantly increase the permeability of the plasma membrane and disturb the photosynthetic system, followed by the deformation of organelles, vacuolization and increasing oxidative stress. The pre-incubation of N-acetyl cysteine (NAC) verified that the rapid damages to the plasma membrane and photosynthetic system caused the algal death in the early phase, and the increasing oxidative stress killed the rest. The late accumulation and possible release of CAT also explained the decreasing toxicity of the algal culture. These results indicated that this microbial algicide has great potential in controlling the growth of *P. globosa* on site.

Marine ecology has attracted increasing attention because of its irreplaceable role in the sustainable development of human society and the maintenance of the earth’s environmental balance^1^. Phytoplankton, as one of the most important primary producers in the ocean, has shown its great influence in the variation of the community structure, energy flow and the geochemical loop^2^,^3^. The abnormal proliferation of some phytoplankton species, also known as harmful algal bloom (HAB), has occurred more frequently in the coastal areas as a result of the increasing nutrient input from land, changing hydrological conditions and a wider spread of HAB-causing species^4^,^5^. HABs not only threaten human health and aquaculture but also destroy the equilibrium of the marine ecosystem. Large accumulations of algal cells shade other phytoplankton, form nuisance foam and deplete oxygen^6^. Various lethal toxins, such as amnesic shellfish toxins, diarrhetic shellfish toxins and paralytic shellfish toxins, are also released to the environment by approximately 80 HAB species^7^. The great biohazards caused by HABs have aroused wide public concern. Many researchers have tried to predict the bloom dynamics using numerical models, which were based on the data collected by monitoring buoys and satellites^8^. Meanwhile, people have never stopped seeking effective methods to prevent or control the HABs^9^.

To reduce the incidence and extent of HABs, controlling the eutrophication of coastal waters is thought to be a good choice^10^,^11^. But once HABs broke out, we should also take some effective actions to quell or contain the blooms before more damages were caused. Nevertheless, only a few techniques, such as clay flocculation^12^, were actually applied to control the HABs on site. Although those methods are efficient in terminating the blooms, there are still some unsolved problems, especially the cost and uncertainty of the eco-safety of these allogenic materials^13^. Therefore, some researchers have turned to the solutions originated from the bloom sites. In the natural waters, microbes can affect the growth of phytoplankton in various ways^14^,^15^,^16^. Some microbes are even the key factors in the natural collapse of HABs, because they can efficiently attack and lyse algal cells^17^,^18^,^19^. The interactions between these microbes and their targeted algae have shown high complexity and diversity, which is closely related with their phylogenetic features. Algal viruses lyse the host cells when the progeny viruses are ready to release^20^. Some bacteria, such as *Cytophaga*^21^, *Saprospira*^22^ and *Roseivirga*^23^, require direct contact with the algal cells before they destroy the cell structures. However, most of the other algicidal bacteria cause algal cell lysis by secreting alleochemicals^24^,^25^,^26^,^27^. These microbial metabolites turn out to be efficient algicides showing great potential in controlling the blooms. But their ecological impact to other aquatic organisms has not yet been well assessed. It seriously hinders the development of this promising method for HAB treatments.

In previous study, we isolated a high efficient algicidal actinomycete *Streptomyces alboflavus* RPS^28^. Its secretion could completely lyse *Phaeocystis globosa* cells within two days. Although data have shown that the growth of many beneficial algae would not be affected by this microbial algicide, it is still necessary to test its ecological safety in more detail and larger scale. Therefore, we established several indoor microcosms to find out the influence of the algicidal process upon the growth of protozoa. We also applied Microtox^®^ test^29^ to precisely monitor the biotoxicity of the microbial algicide and algal exudates. Additionally, considering the toxicity of algal exudates would be closely associated with the intracellular changes of algal cells, we targeted the key metabolisms related to algal death through the following aspects: membrane permeability, cellular ultrastructure, photosynthetic ability and oxidative stress. As a result, this is the first study to closely connect the biotoxicity problem of microbial algicide with its mechanism in lysing the targeted algae. It provides some theoretical evidence that pushes the development of the microbial HAB-control agent.

## Results

### The microbial algicide promoted the growth of protozoa in the microcosms

Testing the algicide in microcosms could not only show its algicidal efficiency in large scale but also reflect its ecological impact to other aquatic organisms. In this study, we applied the microbial algicide from RPS in the 20-L microcosms. The first thing that should be noticed is that the cell density of *P. globosa* in the microcosms (up to 4.91 × 10^8^ L^−1^) was much higher than in the natural bloom areas ((0.5–2) × 10^8^ L^−1^)^30^,^31^,^32^, because the relatively close and stable indoor experimental conditions were beneficial to the massive accumulation of algal cells. Even so, 4 μg/mL of microbial algicide could still significantly reduce the abundance of *P. globosa* after 1d compared to the controls, suggesting the growth of *P. globosa* can be effectively quelled by the microbial algicide (Table 1 and Figure S1). The change of protozoa abundance was also tremendous. In the control groups, both flagellates and ciliates propagated rapidly at day 1. As a result, the abundance of *P. globosa* decreased 24% at day 2, because the free single cells of *P. globosa* were ideal feed for the protozoa^33^. Similarly on this theory, the death of *P. globosa* could provide the protozoa more accessible nutrients due to the massive release of particulate and dissolved organic carbon. Therefore, the abundance of protozoa in the treatment groups even reached a higher level than in the control groups at day 1 and 2. Then, because of the limitation of sufficient nutrient supply (the decrease of *P. globosa* abundance), the abundance of protozoa declined in all experimental groups after day 3, except the flagellates in the control groups. It suggests that the reproduction of protozoa were highly correlated with the availability of food. As for the content of chlorophyll *a*, it decreased along with time, which was matched with the change of *P. globosa* abundance. However, in the treatment groups, the content of chlorophyll *a* maintained at a relatively high level (112.98 ± 0.864 μg/L) at day 5 when the cell density of *P. globosa* had already dropped to only 0.05 ± 0.01 × 10^8^ L^−1^, indicating the existence of other phytoplankton (unidentified in this study). In conclusion, the growth of protozoa and other algae suggests that microbial algicide from RPS would not significantly suppress the growth of other organisms during the lysis of *P. globosa* in a complex experimental system.

### The microbial algicide showed low biotoxicity

**Figure 1:**
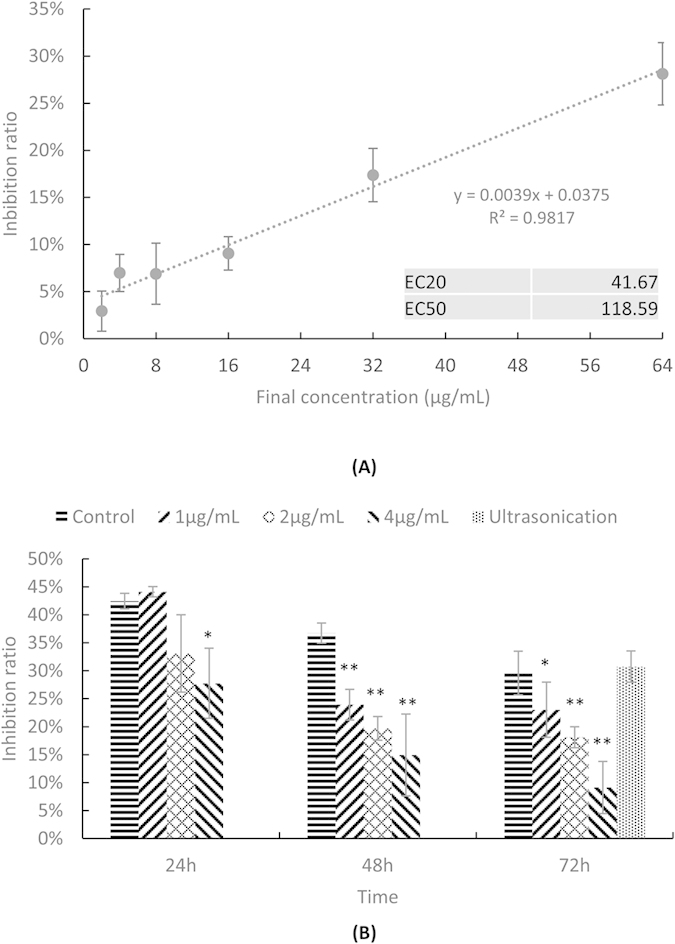
Toxicity of (**A**) different concentrations of microbial algicide, displayed with EC_20_ and EC_50_ values calculated based on the linear regression equation and (**B**) the supernatant of the *P. globosa* culture after being treated with different concentrations of microbial algicide and ultrasonication. Treatments marked with asterisk were significantly different from the control group. * represents 0.01 < *P*-value < 0.05. ** represents *P*-value < 0.01.

Microcosm experiments preliminarily showed the ecological influence of the microbial algicide. To quantify its biotoxicity, we used an approach based on Microtox^®^ to assess the toxicity of seawater samples that were mixed with different concentrations of microbial algicide. In this approach, the inhibition to the luminescent intensity of *Photobacterium phosphoreum* could quantitatively demonstrate the toxicity of the samples, as shown in Fig. 1(A). Based on the data, we successfully established a linear regression equation to calculate the EC_20_ and EC_50_, which were 41.67 and 118.59 μg/mL, respectively. These concentrations were more than 10 times of the highest concentration that was used in the following experiments and microcosms (4 μg/mL). In fact, 4 μg/mL of microbial algicide could only inhibit the luminescent intensity of *P. phosphoreum* by 6.98%, representing negligible toxicity^34^. Thus, the bioluminescent assay suggests that a relevantly high concentration of microbial algicide might exist in the environment safely.

### The microbial algicide could significantly reduce the toxicity of the algal exudates

During its life cycle, *P. globosa* can produce various metabolites^35^, which might be toxic to other marine organisms. The composition of algal exudates could change significantly during the lysis of algal cells. Since the luminescent bacterium *P. phosphoreum* could also characterize the toxicity of the algal culture with different dilution (Figure S2), we used the Microtox^®^ approach again to test the influence of the algicidal process upon the toxicity of the algal exudates [Fig. 1(B)]. Notably, we found out that the treatments with the microbial algicide showed significantly lower toxicity. The reduction of the toxicity of the algal cultures was positive correlative with concentration and processing time of the microbial algicide. After exposing the algae to 4 μg/mL of microbial algicide with 72 h, the inhibition ratio dropped to only 9.14%, representing a nontoxic algal culture. To understand whether it was a common phenomenon caused by algal lysis, we also completely killed the algal cells using ultrasonication. However, this physical approach showed no effect on the toxicity. It suggests that the microbial algicide might induce a unique death pathway in algal cells, which leads to the release of some different metabolites that neutralize the toxins in algal exudates.

### The microbial algicide would instantly affect the permeability of the cellular membrane

**Figure 2:**
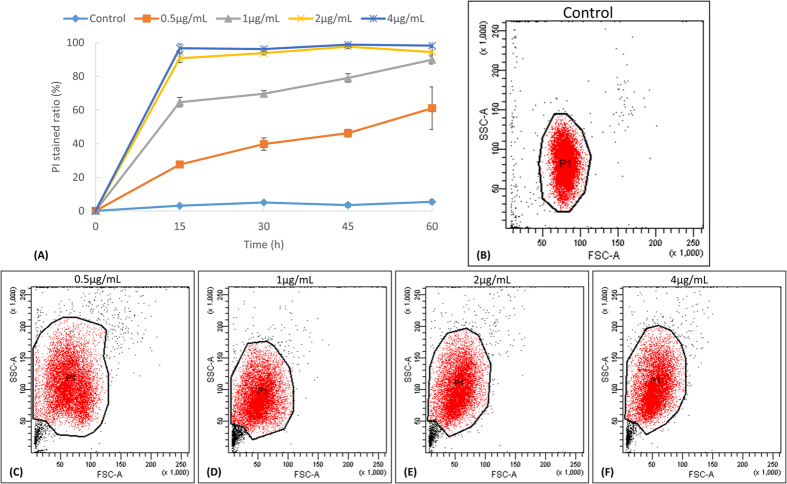
(**A**) The percentage of PI stained *P. globosa* cells after being treated with different amounts of microbial algicide for different amounts of times. Scatter plots represent the volume (FSC) and complexity (SSC) of algae after 1 h of treatment. (**B**) Control, (**C**) 0.5 μg/mL, (**D**) 1 μg/mL, (**E**) 2 μg/mL, (**F**) 4 μg/mL.

Because there is no cell wall covering on the surface of *P. globosa*, the proper functioning of the plasma membrane is essential for maintaining the cellular structure and normal metabolisms. Here we used presidium iodide (PI) as the indicator of membrane permeability. PI would not penetrate the plasma membrane of healthy algal cells and stain the nucleus [Fig. 2(A) and S3(A)]. However, even a very small dosage of microbial algicide would rapidly and severely change the permeability of most algal cells after 1h [Fig. 2(A) and S3]. Higher concentrations of microbial algicide also led to more PI-stained cells in shorter time [Fig. 2(A)]. Such significant change of permeability in a short time suggestes that the microbial algicide may directly act on the cellular membrane. There is no doubt that it would trigger a series of irreversible damage and stress response in the algal cells.

The change of membrane permeability was also corroborated by the cell volume and cell complexity, which appeared as forward-scattered light (FSC) and side-scattered light (SSC) in the flow cytometer. With the higher concentration of microbial algicide, the majority of algal cells became smaller (lower FSC) and showed higher complexity inside (higher SSC) [Fig. 2(B)~(F)]. Because the microbial algicide greatly affected the permeability of membrane, the hypertonic seawater environment could easily cause the shrink of algal cells. As for the cellular complexity, it was usually correlated with intracellular particles or vacuoles. A higher level of complexity could be interpreted as the changes in cellular ultrastructure and severe damage to organelles. To visualize the changes in the cellular ultrastructure, we then used transmission electron microscopy (TEM) to observe the algal cells at different processing times.

### The algal cells demonstrated severe cytoplasmic vacuolization

**Figure 3:**
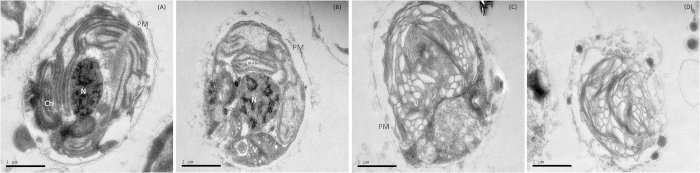
Ultrastructure of *P. globosa* after treatment with 4 μg/mL of microbial algicide. (**A**) Control, (**B**) 4 h, (**C**) 12 h, (**D**) 24 h. Abbreviations: N: nucleus Ch: chromatophore PM: plasma membrane V: vacuole.

TEM (Fig. 3) revealed the deformation of the cellular ultrastructure of *P. globosa* cells gradually toward death. Figure 3(A) shows a healthy *P. globosa* cell which has the intact plasma membrane, the tightly stacked lamellar structure of the chromatophore, and a nucleus in good shape. However, after 4 h of incubation with the microbial algicide, the distortion of organelles and nucleus could be observed, as shown in Fig. 3(B). To be specific, the plasma membrane began to degrade. The lamellar structure became loose. The nucleus appeared in an irregular shape. Meanwhile, some small vacuolization could be noticed in partial area of the cytoplasm. Vacuolization became serious and was widely distributed in the cytoplasm after 12 h of incubation [Fig. 3(C)]. The organelles and nucleus were also difficult to distinguish, but cell inclusions seemed to be integral at this time point. After 24 h of incubation, the plasma membrane ruptured, which left almost only an empty shell, indicating the final death of algal cells [Fig. 3(D)]. Throughout the whole process, we could see that the breakdown of organelles and vacuolization were important signs of cell lysis. Predictably, the normal function of these organelles would be severely damaged.

### The photosynthetic system was rapidly damaged by the microbial algicide

**Figure 4:**
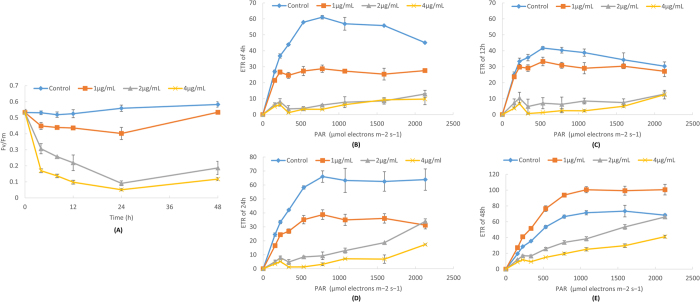
Photosynthetic efficiency (Fv/Fm) (**A**), photosynthetic capacity (rETR) after treatment with microbial algicide for 4 h (**B**), 12 h (**C**), 24 h (**D**) and 48 h (**E**).

For a photoautotroph, photosynthesis is the source of energy, organic matters and oxygen, which makes it one of the most active and crucial metabolisms in the algal cells. Considering that the microbial algicide could instantly change the permeability of the membranes, the biochemical interactions happened in the chromatophores might be easily interfered. It suggests that the photosynthetic system would suffer severe damage. This speculation was preliminary verified by the TEM images. Figure 3 had showed that the lamellar structure of chromatophores broke down from the very beginning of the death process. We then use a phyto-PAM to directly measure the photosynthetic efficiency and capacity of *P. globosa*. Treated with high concentrations of microbial algicide (2 and 4 μg/mL), the photosynthetic efficiency (Fv/Fm) fell to a very low level within just 4 h [Fig. 4(A)]. The photosynthetic capacity (rETR) of these treatments was also significantly lower than that of the control group [Fig. 4(B)]. Throughout the first 24 h, high concentrations of the microbial algicide could almost completely shut down the photosynthesis. However, the photosynthetic ability seemed to begin to recover at 48 h. The Fv/Fm value was more or less raised [Fig. 4(A)] and the ETR curve of the 1 μg/mL treatment was even higher than that of the control group [Fig. 4(E)]. This finding could be explained by the self-repairing mechanism of PS II. It also suggested that the microbial algicide might have been degraded and no longer suppress the surviving cells after 48 h. Because most algal cells have been lysed at this time, the potential degradable property of microbial algicide decreases its danger in the environment without compromising the algicidal efficiency.

### Oxidative stress was an important cause of algal cell death

**Figure 5:**
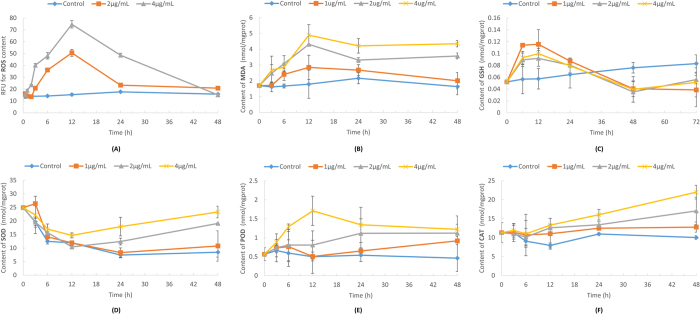
Effects of the microbial algicide on the content of ROS (**A**), MDA (**B**), GSH (**C**), SOD (**D**), POD (**E**) and CAT (**F**).

One of the byproducts of photosynthesis is ROS, especially when the photosynthetic system is not functioning normally. The excess light energy induces the overproduction of ROS that causes the huge stress to all organelles. To demonstrate the extent of oxidative damage and the response of the antioxidant system, we measured the content of ROS, MDA, SOD, POD, CAT and GSH (Fig. 5) in the algal cells during the whole algicidal process. Previous researches^36^,^37^,^38^ have revealed the mechanism concerning how cells handle oxidative stress. GSH and other non-enzyme antioxidants could act as the “quick reaction force” and remove the excess ROS. Once the ROS content was beyond the buffering capacity of the GSH pool, the production of antioxidant enzymes was then regulated by the signalling pathway, so that the enzymes could neutralize the residual ROS. Our data matched this mechanism when the algal cells were treated with 1 μg/mL of microbial algicide. In this treatment, the extent of oxidative damage demonstrated by the content of MDA [Fig. 5(B)] was relatively low (MDA is the final product after the ROS attacked the polyunsaturated fatty acid in the membrane and induced lipid peroxidation). Therefore, only the content of GSH reached a high level to resist the minor oxidative stress, whereas the content of antioxidant enzymes (POD, CAT and SOD) maintained at the same level as the control group.

Higher concentrations of microbial algicide would show a different pattern. As shown in Fig. 5(A), the ROS content started increasing at 2 h, reached its peak at 12 h and fell back to a low level after 24 h. The variation trend of oxidative stress was consistent with the production and consumption of GSH. The GSH content reached its peak at approximately 12 h and rapidly became lower than the control group [Fig. 5(C)]. However, judging by the MDA content [Fig. 5(B)], the oxidative damage was still high, suggesting GSH could not eliminate the stress of ROS in time. In this case, the antioxidative mechanism should instantly upregulate the synthesis of antioxidant enzymes. It was just partly correct in this study. Only the POD content [Fig. 5(D)] rapidly reached a high level at 12 h when the algae was treated with 4 μg/mL of microbial algicide. However, the curves representing the SOD and CAT contents in the treatment groups deviated from the control group slowly and only showed significant differences after 24 h (*P*-value < 0.01) [Fig. 5(E,F)]. It explains the continuously high MDA content in these experimental groups. Thus, high oxidative stress induced by the microbial algicide could be an important cause of death for the algal cells.

**Figure 6:**
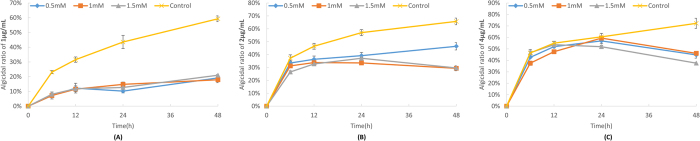
Effects of NAC on the algicidal ratio when the algae were treated with 1 μg/mL (**A**), 2 μg/mL (**B**) and 4 μg/mL (**C**) of microbial algicide.

To see the connection between oxidative stress and algal cell death, we also pre-incubated the algal cells with different concentrations of NAC before microbial algicide was added. NAC is a widely used ROS scavenger that can reduce the oxidative stress. As would be expected, NAC was effective in saving the algae from the cell death induced by the microbial algicide, particularly when the concentration of microbial algicide was low [Fig. 6(A)]. However, the oxidative stress was just partially responsible for the death of algal cells. With the increasing concentration of microbial algicide, NAC gradually had no effect on the algicidal ratio at the early phase [Fig. 6(B,C)]. But it could still help the survival of algae after certain time points, depending on the concentrations of microbial algicide. Considering that the damaged plasma membrane and chromatophore could be the primary cause of the algal death at the early phase, our study also suggested that the increasing oxidative stress might be responsible for the death of the surviving algal cells at the late phase.

### CAT could decrease the toxicity of algal culture

**Figure 7:**
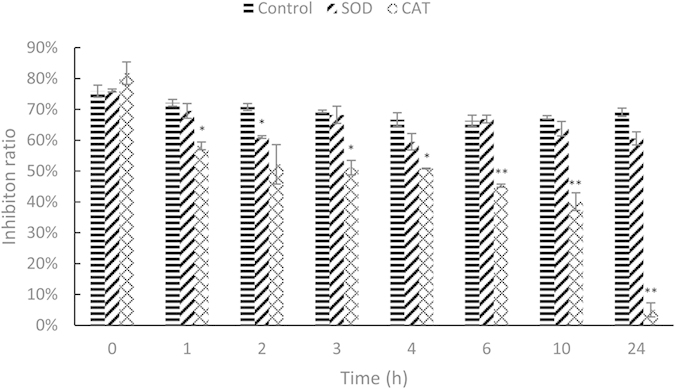
Toxicity of the *P. globosa* culture after being incubated with CAT and SOD (500 U/mL). Treatments marked with asterisk(s) were significantly different from the control group. * represents 0.01 < *P*-value < 0.05. ** represents *P*-value < 0.01.

As shown in Fig. 5(E,F), the contents of SOD and CAT were high during the late phase of algal death. Because they would be released to the environment along with the breakdown of the plasma membrane, they might act as the antidotes against the toxins in algal exudates. To confirm this speculation, we tested the toxicity of the algal supernatant (no algal cells) that was remediated with SOD and CAT. The results showed that the addition of CAT started to decrease the toxicity of the algal supernatant after only 1 h. The inhibition effect of the supernatant continued to decline, until a negligible level at 24 h (Fig. 7). These data suggest the important role of CAT in improving the water quality of the algal culture. In other words, although SOD had almost no significant effect on the toxicity of the algal supernatant, the release of these antioxidant increased the ecological safety of the microbial algicide virtually.

## Discussion

Taking full advantage of the microbe-alga interaction to prevent or terminate HABs is a promising but unverified way to eliminate the harms of these blooms^4^. However, the initial goal of environmental protection would naturally lead to concerns about the ecological safety of such microbial algicides. Microcosms are commonly used in the assessment of algicide^13^,^39^,^40^. In this study, we established the microcosms to investigate the algicidal efficiency of microbial algicide and its impact on heterotrophic protozoa. Unlike the natural environment, microcosms can provide a stable and favourable condition for the growth of *Phaeocystis globosa*. Therefore, the algal density could reach an extremely high level (up to 4.91 × 10^8^ L^−1^). Theoretically, the population of *P. globosa* should then descend since there was no subsequent nutrient supply to fulfil the high consumption under this circumstance. However, even though the predation of ciliates and heterotrophic flagellates also greatly promote the mortality of algae, the decline of *P. globosa* was still slow in the control microcosms (approximately 20% in 5 days). It implied the indomitable vitality of *P. globosa*, which might cause long-term pollution in the ocean. Nevertheless, the addition of microbial algicide could rapidly remove these harmful algae within two days. Admittedly, the concentration of microbial algicide we used (4 μg/mL) is relatively high for the microcosm experiments, because this amount of algicide can easily kill the algae in a higher density (for example, 10^9^ L^−1^, which was used in the following small-scale experiments). Even so, such high concentration of the microbial algicide would not significantly inhibit the growth of the protozoa and other phytoplankton. The explosive growth of protozoa in the treatment groups could be partially explained by the increasing availability of nutrients. Another possible reason was that the stress caused by the harmful algae had been eliminated. This speculation was confirmed by the bioluminescent assays. Firstly, the biotoxicity of the microbial algicide was very low. Secondly, the toxicity of algal exudates was also significantly decreased after the algal cells were lysed by the microbial algicide. It suggests that the living condition for the other aquatic organisms was getting better during the lytic process. All these results support the conclusion that removing the harmful algae using microbial algicide was conducive to the remediation of the bloom waters.

**Figure 8:**
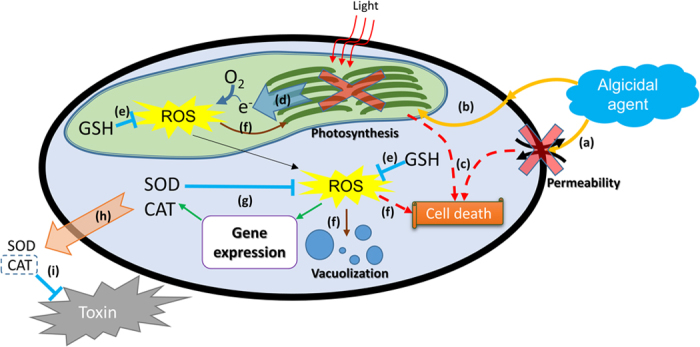
The possible mechanism of how the microbial algicide produced by *S. alboflavus* RPS induced the lysis of *P. globosa*. (**a**)~(**i**) indicate different stages in the death process. Details were described in the article.

The assessment of ecological safety was then supported by the exploration of algicidal pathway of microbial algicide. Figure 8 summarizes the possible mechanism of *P. globosa* lysis: (a) The algicidal compound might firstly influence the material exchange system on the plasma membrane. (b) This effect quickly spread to other organelles, such as the photosynthetic system, which was highly dependent on a properly functioning membrane. (c) If the concentration of microbial algicide was high or the algal cells were vulnerable, the lysis of cells would occur. (d) If the algal cells survived the early stage, they would also suffer the high oxidative stress originating from the abnormal photosynthetic system. (e) GSH was consumed constantly to clean the ROS. (f) Meanwhile, the other antioxidant enzymes, such as SOD and CAT, were unable to relive the oxidative stress in time. (g) Therefore, vacuolization was caused; organelles were damaged continuously. All these led to the final death of algal cells. This hypothetical mechanism could also explain the decreasing toxicity of the algal culture after algal cells were lysed by the microbial algicide. The antioxidants (SOD and CAT in this study) were highly expressed at the very late phase of algal death. However, cell death was already irreversible in this time. Because of the increasing permeability of the plasma membrane and the rapid rupture of the cells, these antioxidants were released to the environment [Fig. 8 (h)]. Once these antioxidants existed in the algal culture, CAT might have acted as the antidote by scavenging the free radicals or helping the other organisms resist the oxidative stress induced by the toxins [Fig. 7 (i)]; either way, the toxicity of the algal culture ultimately appeared to be much lower.

To summarize, this study comprehensively revealed the ecological influence and the algicidal pathway of a microbial algicide produced by an actinomycete, which enriches the theories of microbe-alga interactions that have attracted increasing interest and attention. At the macro level, these interactions can be linked to the food web or biological pump, which greatly influences the eco-system and the biogeochemical cycle^41^. At the micro level, these interactions are involved with multiple important metabolism pathways in microbes or algae^42^. It also inspires the idea of using the microbial algicide to control the HABs, or remove the unwanted algae in the photobioreactors. However, this method is still not applicable to any outdoor ecosystem at the present stage. More efforts are still needed to optimize the application of microbial algicide. Firstly, the dosage of the algicide should be carefully tuned to match extent of the algal blooms and the oceanic conditions. Secondly, some bioaugmentation technologies should be utilized during the field application in order to reduce the loss of microbial algicide and prolong the effective time. Thirdly, the negative effect of microbial algicide on another aquatic organisms, such as molluscs and fishes, should also be well tested. Finally, more transcriptome, proteome and metabolome information is still required to systematically reveal the changes of every metabolism pathway in algal cells. All these are the directions of our future efforts. We hope HABs could be ultimately quelled and contained in an efficient and secure way.

## Methods

### Cultivation of the actinomycete and alga

The algicidal actinomycete we studied was previously identified as *Streptomyces alboflavus* RPS (MCCC 1F01215). The strain was initially cultivated with a modified Gause medium (soluble starch 15 g/L, NaNO_3_ 1 g/L, K_2_HPO_4_ 0.5 g /L, MgSO_4_•7H_2_O 0.5 g/L, FeSO_4_•7H_2_O 0.01 g/L, dissolved in deionized water), in a rotary shaker (28 °C, 200 rpm) for 6d until the mycelia turned yellow-red^28^.

The *Phaeocystis globosa* culture was obtained from the State Key Laboratory of Marine Environmental Science (Xiamen University). The algal culture was transferred weekly to a fresh sterilized f/2 medium and maintained under a 12 h: 12 h light-dark cycle with a light intensity of 4000 lx at 20 ± 1 °C.

### Extraction of microbial algicide

To increase the production of the algicidal compound, we transferred the strain to a well-optimized medium (soluble starch 17.76 g/L, NaNO_3_ 1.59 g/L, K_2_HPO_4_ 0.5 g/L, MgSO_4_•7H_2_O 0.5 g/L, FeSO_4_•7H_2_O 0.01 g/L, pH 5.23) with an inoculum size of 8.1% and a fermentation time of 185 h^43^. Collecting 5 L of fermentation broth, we filtrated out the mycelia and vacuum evaporated the broth to 500 mL. The broth was then extracted with an equal volume of ethyl acetate three times. The extract was primarily separated with a Sephadex^TM^ LH-20 column. Each fraction was evaporated and weighed in order to dissolve approximately 20 μg in dimethyl sulfoxide (DMSO). Five μg of each fraction was tested for the algicidal ratio (Supplementary Information S1) to finally determine the effective algicidal fraction, of which the algicidal ratio was above 80%. These fractions were then mixed together, evaporated and stored in a solid state, and this was the microbial algicide used in the following experiments. Before each experiment, the microbial algicide was dissolved in DMSO to a final concentration of 10 μg/μL.

### Microcosm experiments

Evaluation of the algicidal effect and ecological safety of the microbial algicide was conducted in indoor microcosms. 20 L of seawater for each microcosm was filtered through a 0.45 μm polycarbonate membrane (Millipore) and contained in a 50-L plastic enclosure. The seawater was amended with the same nutrients as the f/2 medium and used to incubate *Phaeocystis globosa* under illumination of 1000 lx (12 h: 12 h light-dark cycle) at 20 °C. When the density of algal cells reached the bloom level (approximately 10^8^ L^−1^), natural protozoa and phytoplankton (unidentified, mostly *Skeletonema costatum*) collected from Yundang Lake (a seawater lake in Xiamen Island) were added to each microcosm (heterotrophic flagellates ≈ 10^6^ L^−1^, ciliates ≈ 10^4^ L^−1^). 4 mg/L of microbial algicide was then added into the microcosms, while the same volume of DMSO (20 mL) was added into the other microcosms as controls. The growth of phytoplankton and protozoan was measured at 1-day intervals for a period of 5 days. The cell densities of *P. globosa* and population compositions of protozoa were determined microscopically using appropriate counting chambers. The biomass of phytoplankton was determined by the concentration of Chl *a*. Samples from each microcosm was filtered through a GF/F filter (Whatman) and extracted with 95% ethanol. *In vivo* fluorescence was measured using a microplate reader (Spectra max M2, Molecular Devices Corporation). All experiments were performed in triplicate.

### Bioluminescent assay

To test the toxicity of the microbial algicide, it was added to the experimental tubes with 1.5 mL fresh seawater to final concentrations of 2, 4, 8, 16, 32 and 64 μg/mL. The inhibition ratio of each concentration was tested using the modified Microtox^®^ approach (Supplementary Information S2). The EC_20_ and EC_50_ values were calculated based on the linear regression equation.

To check whether the bioluminescent assay could quantify the toxicity of the *P. globosa* exudates, we centrifuged the culture (growing for 14 d, late steady phase) at 4,000 rpm for 10 min (Eppendorf-5424R) to remove the algal cells. The supernatant was then diluted with a fresh f/2 medium relevant for the dilution factors of 0.2, 0.4, 0.6 and 0.8. Using the fresh f/2 medium as the control, the luminescent inhibition caused by each concentration of the algal supernatant was tested using the approach described in Supplementary Information S2.

To determine the toxicity change of the algal exudates during algal cell lysis by the microbial algicide, algal cultures were added with 1, 2 and 4 μg/mL of microbial algicide and sampled of 24, 48 and 72 h. We also lysed the algal cells using ultrasonication (Ultrasonic Cell Disruption System by NingBo Scientiz Biotechnological Co., Ltd, China; 100 W, 5 s working with 5 s gaps, 55 cycles, ice bath) to compare the toxicity of the algal culture the algal cells were lysed through physical versus biological methods.

### Flow cytometry

To monitor the permeability of the plasma membrane, we used propidium iodide (PI) to stain the algal cells and examined them using flow cytometry. In the experimental setup, we treated the algal cultures with 0, 0.5, 1, 2 and 4 μg/mL of microbial algicide and sampled at 0, 15, 30, 45 and 60 min. The algal cells were collected, washed with PBS (0.1 M, pH7.4) and inoculated with 0.1 mg/mL PI for 30 min at dark. The cells were then examined for forward-scattered light (FSC), side-scattered light (SSC) and PI fluorescence using a BD LSR FORTESSA cell analyser. The data were further analysed using BD FACSDiva software.

### Transmission electron microscopy

Here we incubated the algal culture with 4 μg/mL of microbial algicide and sampled at 0, 4, 12, and 24 h. The algal cells were collected and fixed overnight at 4 °C in 0.1 M PBS containing 2.5% glutaraldehyde (v/v). The cells were then dehydrated through a graded ethanol series (30, 50, 70, 90 and 100% (v/v)) and then a graded ethanol:acetone series (3:1, 1:1, 1:3, 0:1) at 4 °C. Embedded in araldite resin and sliced with an ultramicrotome, the sections (60–80 nm) were stained in 3% acetic acid uranium-citric acid and viewed using a JEM2100HC (Japan) transmission electron microscope^44^.

### Phyto-PAM

A PAM-CONTROL Fluorometer (Walz, Effeltrich, Germany) was used to measure the maximum quantum yield (Fv/Fm) and the relative electron transport rate (rETR/μmol electrons m^−2^ s^−1^). In this experiment, the final concentrations of microbial algicide in each experimental group were 1, 2 and 4 μg/mL and the sampling time points were 4, 8, 12, 24 and 48 h. The algal cultures were dark-adapted for 15 min and measured for Fv/Fm under a strong, red LED (>3000 μmol m^−2^ s^−1^)^45^, and rETR measurement followed. Eight consecutive light levels of 156, 226, 337, 533, 781, 1077, 1593 and 2130 μmol photons m^−2^ s^−1^ were applied at 15 s intervals^46^. Fv/Fm and rETR were the main indicators for the photosystem II efficiency and for the photosynthetic capacity.

### Cellular inclusion assays concerning oxidative stress

We first measured the intracellular ROS content using DCFH-DA (Beyotime). In this experiment, 2 and 4 μg/mL of microbial algicide was added to the algal cultures and sampling time points were 0.5, 1, 2, 3, 6, 12, 24 and 48 h (0.5, 6, 12, 24 and 48 h for control group). The algal cells were collected, washed with a fresh f/2 medium, and inoculated with 10 μM DCFH-DA for 30 min at 37 °C. The cells were then washed three times and resuspended in the medium until OD_680_ was approximately 0.8. Finally, 200 μL of the cell suspension was added to a 96-well plate and measured for the RFU under an excitation wavelength of 488 nm and an emission wavelength of 525 nm. The fluorescence intensity was positively related to the intracellular ROS content.

We then measured lipid peroxidation and several antioxidants using commercial assay kits, including those for total protein quantification (Coomassie brilliant blue), malondialdehyde (MDA; the thiobarbituric method), reduced glutathione (GSH; spectrophotometric method), peroxidase (POD), superoxide dismutase (SOD; WST-1 method) and catalase (CAT; visible light). All kits were manufactured by the Nanjing Jiancheng Bioengineering Institute, China. The final concentrations of microbial algicide used to treat the algae were 1, 2 and 4 μg/mL, and the sampling time points were 0, 3, 6, 12, 24 and 48 h (0, 6, 12, 24, 48 and 72 h in GSH measurement). The algal cells were disrupted using ultrasonication (under the same conditions as described in bioluminescent assay), and debris was removed using centrifugation at 10,000 rpm for 10 min at 4 °C. The subsequent measurement and data analysis were conducted following the operational manuals of the kits^47^.

We also tested the change in the algicidal ratio when the algal cells were pre-inoculated with N-acetyl cysteine (NAC), which was first dissolved in a fresh f/2 medium to 100 mM and then added to the algal culture at the final concentrations of 0 (control), 0.5, 1, and 1.5 mM. After 1 h inoculation, microbial algicide was added to the algal culture. The final concentrations of microbial algicide were 1, 2 and 4 μg/mL, and the sampling time points were 0, 6, 12, 24 and 48 h. The algicidal ratio was calculated as described in Supplementary Information S1.

### Verification of the effects of SOD and CAT

Suspecting the potential effects of SOD and CAT in detoxification, we tested the toxicity of the algal culture that was incubated with the commercialized SOD and CAT (Beyotime). The supernatant of the algal culture was collected as described above. The powder of SOD and CAT was first diluted with ddH_2_O and added to the supernatant of the algal culture with a final concentration of 500 U/mL (v/v = 1%). The control group was added to the same volume of ddH_2_O. After 0, 1, 2, 3, 4, 6, 10 and 24 h of incubation at 20 °C, the toxicity of the supernatant was tested with the method described in Supplementary Information S2.

### Data processing

All data were organized and calculated by Excel 2013 and SPSS 20.0.

## Additional Information

**How to cite this article**: Cai, G. *et al*. Lysing bloom-causing alga *Phaeocystis globosa* with microbial algicide: An efficient process that decreases the toxicity of algal exudates. *Sci. Rep*. **6**, 20081; doi: 10.1038/srep20081 (2016).

## Supplementary Material

Supplementary Information

## Figures and Tables

**Table 1:** Summary of changes in biotic factors in *P. globosa*-inoculated microcosms.

Items	Day 0		Day 1		Day 2		Day 3		Day 4		Day 5	
	Control	Algicide	Control	Algicide	Control	Algicide	Control	Algicide	Control	Algicide	Control	Algicide
*P. globosa*(×10^8^/L)	4.30 ± 0.42	4.91 ± 0.37^NS^	4.84 ± 0.58	2.59 ± 0.43*	3.68 ± 0.34	0.38 ± 0.14**	3.35 ± 0.12	0.13 ± 0.05**	3.26 ± 0.29	0.08 ± 0.01**	3.34 ± 0.38	0.05 ± 0.01**
Ciliates(×10^4^/L)	5.33 ± 1.25	6.33 ± 1.70^NS^	11.67 ± 3.86	18.33 ± 0.94^NS^	12.75 ± 5.12	39.67 ± 8.34*	9.25 ± 5.31	28.00 ± 5.79*	5.67 ± 2.36	30.25 ± 7.19*	8.33 ± 3.86	21.00 ± 5.10*
Flagellates(×10^6^/L)	1.23 ± 0.24	1.40 ± 0.21^NS^	2.66 ± 0.12	6.00 ± 0.59**	3.52 ± 0.43	8.58 ± 0.71**	5.28 ± 0.49	4.66 ± 0.33*	5.42 ± 0.92	4.11 ± 0.54*	6.14 ± 0.19	6.21 ± 0.30^NS^
*In vivo*Fluorescence	58.52 ± 2.28	56.97 ± 4.03^NS^	47.53 ± 2.37	21.24 ± 1.04**	46.13 ± 3.56	6.33 ± 1.30**	36.87 ± 0.72	5.49 ± 0.81**	36.44 ± 1.68	4.57 ± 0.47**	35.97 ± 2.87	4.63 ± 0.75**
Chl *a*(μg/L)	368.28 ± 2.83	381.50 ± 6.76^NS^	325.23 ± 2.41	279.90 ± 2.45**	276.91 ± 5.19	156.85 ± 3.24**	257.69 ± 1.93	134.21 ± 1.05**	220.42 ± 1.03	122.54 ± 0.62**	209.12 ± 1.62	112.98 ± 0.86**

Data show the mean ± SD.

The differences between the control and algicide groups were judged by the *P*-value. NS: not significant. *: 0.01 < *P*-value < 0.05. **P-value < 0.01.
