# Supplementary material for: Lysing bloom-causing alga Phaeocystis globosa with microbial algicide: An efficient process that decreases the toxicity of algal exudates
Source: Sci Rep. 2016 Feb 5;6:20081. doi: 10.1038/srep20081 (PMC4742815; doi:10.1038/srep20081)
Supplement: Supplementary Information [file srep20081-s1.doc]

**Supplementary information**

Authors: Guanjing Cai, Xujun Yang, Qiliang Lai, Xiaoqi Yu, Huajun Zhang, Yi Li, Zhangran Chen, Xueqian Lei, Wei Zheng, Hong Xu, Tianling Zheng*

Title: Lysing bloom-causing alga *Phaeocystis globosa* with microbial algicide: An efficient process that decreases the toxicity of algal exudates

**S1 The calculation of algicidal ratio**

The algal biomass was evaluated by measuring the relative fluorescent units (RFU) under an excitation wavelength of 440 nm and an emission wavelength of 680 nm (Spectra max M2, Molecular Devices Corporation).

To see the algicidal activity, the algicidal fraction was added to 1 mL of *P. globosa* culture in a 24-well plate (initial RFU approximately = 300), and the same amount of DMSO was added to algal culture as a control. After 48 h, the algicidal ratio of each fraction was calculated using the following formula:

Algicidal ratio (%) =
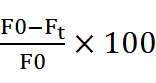
,

where Ft is the fluorescent intensity of the treated algal culture, and F0 is the fluorescent intensity of the control group.

All fractions were tested three times.

**S2 The detailed procedure of bioluminescent assay**

We made several modifications to the original Microtox® approach to fit our experimental setup and better test the toxicity of the algicidal extract and algal culture.

The luminescent bacterium *Photobacterium phosphoreum* (used in the Chinese national standard protocol GB/T 15441-1995) was stored at -80°C and activated with a bouillon peptone medium containing 3% NaCl and 1% glycerin in a rotary shaker (28°C, 200 rpm) for 18-24 h. To see whether the bacterium was well activated and suited for the following toxicity monitoring, 5 μL of the culture was added to 1.5 mL of fresh f/2 medium and the relative luminescent intensity (RLU) was measured with a Berthold Sirius L Luminometer. The RLU remained relatively steady between 5,000,000 and 6,000,000 for 20 min (Figure S4), which was the standard growth status of *P. phosphoreum* used in the following bioluminescent assay.

During the experiments, each experimental tube, which contained a 1.5 mL water sample, was coupled with a control tube containing the same 1.5 mL fresh seawater. Five μL of the *P. phosphoreum* culture was added to each tube and measured for the RLU after a precisely 15 min reaction (Figure S4). The inhibition ratio of the luminescence intensity was calculated using the following formula:

Inhibition ratio (%) =
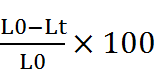
,

where Lt is the luminescence intensity of the experimental tube, and L0 is the luminescence intensity of the control tube.

All experimental groups were replicated three times.


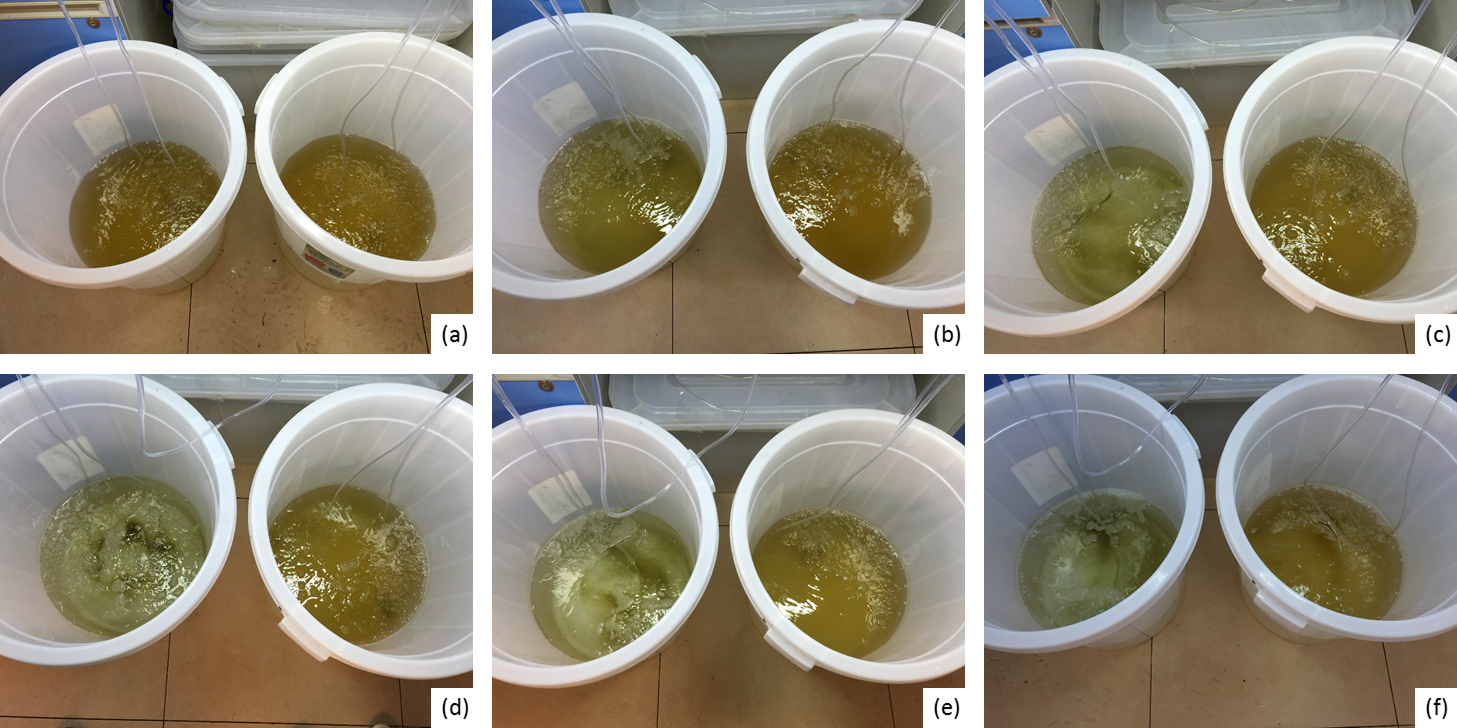


**Figure S1** Microcosms that simulated the *P. globosa* blooms. The left one was treated with the microbial algicide while the right one was the control. (a) to (f) show the pictures taken at different time (Day 0-5).


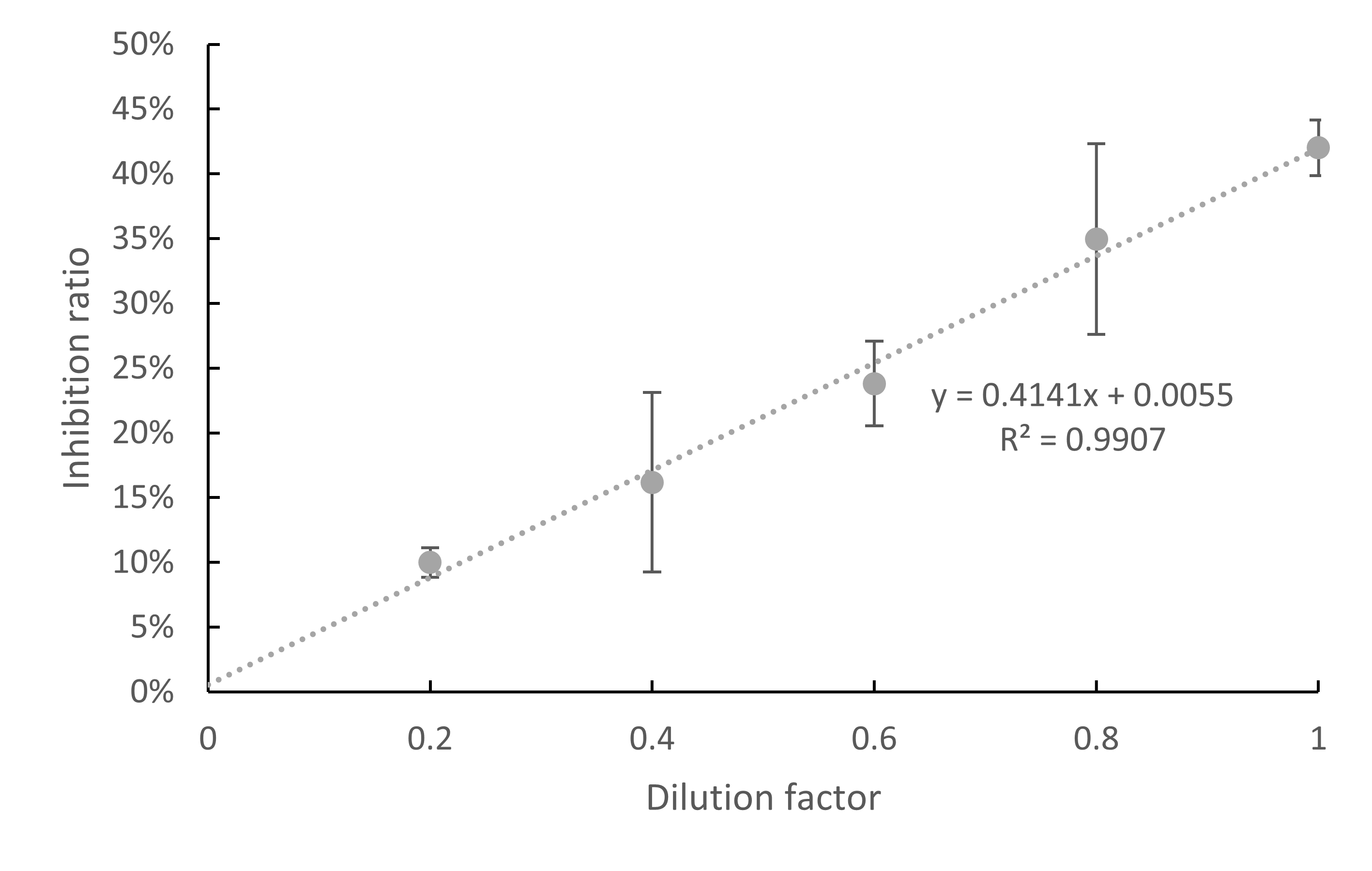


**Figure S2** Toxicity of the supernatant of the *P. globosa* culture after being diluted with a graded series. The original supernatant of the algal culture showed quite strong toxicity and inhibited the luminescent intensity by 42.02%. With different dilutabilities, the inhibition ratio exhibited an excellent linear decrease, which suggested that *P. phosphoreum* was also fit for monitoring the eco-toxicity of the algal culture.


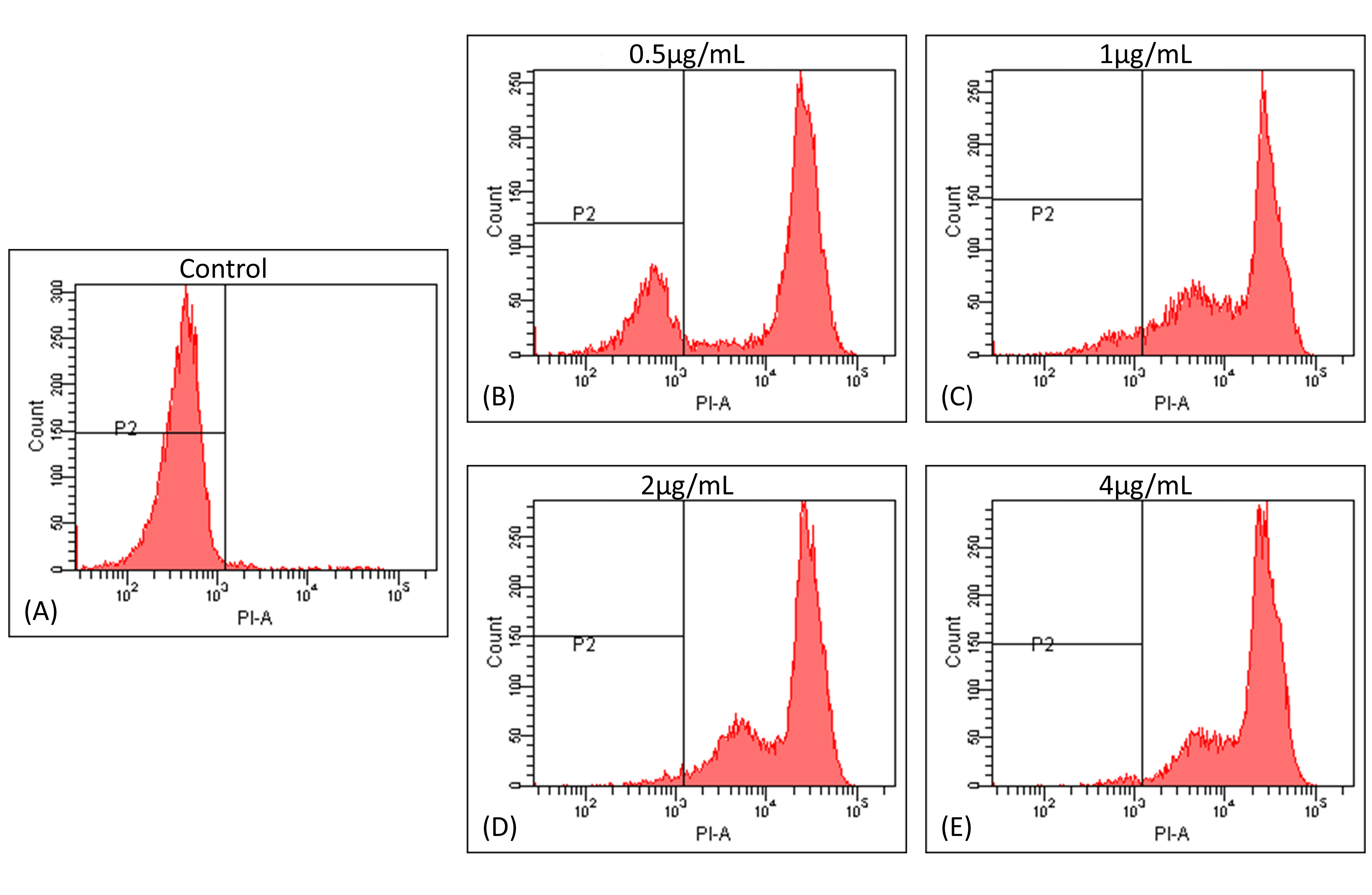


**Figure S3** Algal cells stained with PI, after being treated with different amounts of algicidal extract for 1 h. (A) Control, (B) 0.5 µg/mL, (C) 1 µg/mL, (D) 2 µg/mL, (E) 4 µg/mL.


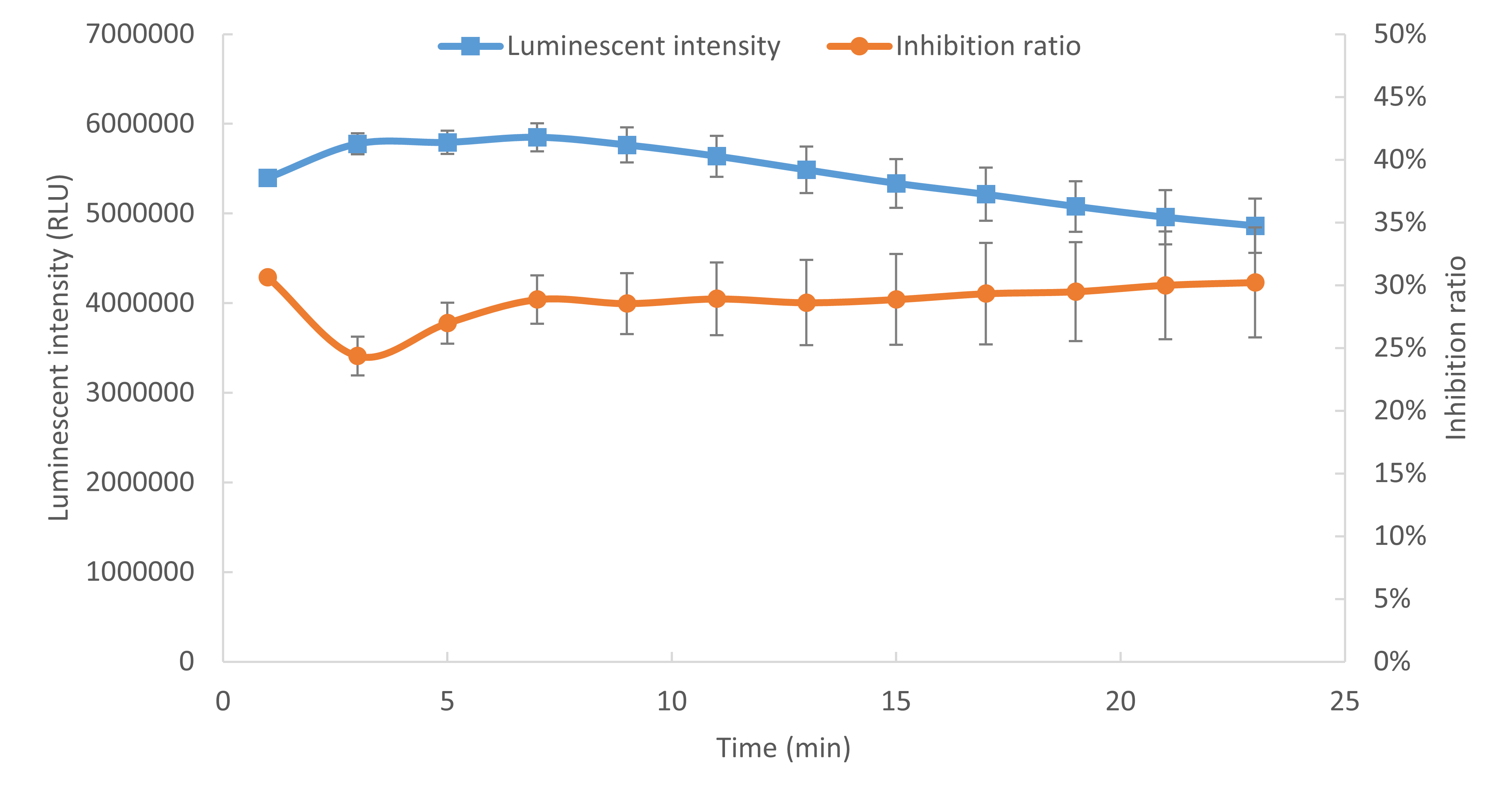


**Figure S4** Along the reaction time, the luminescent intensity of *P. phosphoreum* in the control tubes, as well as the inhibition ratio of experimental tubes when tested with the supernatant of *P. globosa*.
